# Impact of Mean Annual Temperature on Nutrient Availability in a Tropical Montane Wet Forest

**DOI:** 10.3389/fpls.2020.00784

**Published:** 2020-06-12

**Authors:** Creighton M. Litton, Christian P. Giardina, Kristen R. Freeman, Paul C. Selmants, Jed P. Sparks

**Affiliations:** ^1^Department of Natural Resources and Environmental Management, University of Hawai‘i at Mānoa, Honolulu, HI, United States; ^2^Institute of Pacific Islands Forestry, Pacific Southwest Research Station, USDA Forest Service, Hilo, HI, United States; ^3^Western Geographic Science Center, United States Geological Survey, Menlo Park, CA, United States; ^4^Department of Ecology and Evolutionary Biology, Cornell University, Ithaca, NY, United States

**Keywords:** ecological stoichiometry, Hawaii, litterfall, macro- and micronutrients, mean annual temperature, nutrient resorption efficiency, nutrient use efficiency

## Abstract

Despite growing understanding of how rising temperatures affect carbon cycling, the impact of long-term and whole forest warming on the suite of essential and potentially limiting nutrients remains understudied, particularly for elements other than N and P. Whole ecosystem warming experiments are limited, environmental gradients are often confounded by variation in factors other than temperature, and few studies have been conducted in the tropics. We examined litterfall, live foliar nutrient content, foliar nutrient resorption efficiency (NRE), nutrient return, and foliar nutrient use efficiency (NUE) of total litterfall and live foliage of two dominant trees to test hypotheses about how increasing mean annual temperature (MAT) impacts the availability and ecological stoichiometry of C, N, P, K, Ca, Mg, Mn, Fe, Zn, and Cu in tropical montane wet forests located along a 5.2°C gradient in Hawaii. Live foliage responded to increasing MAT with increased N and K concentrations, decreased C and Mn concentrations, and no detectable change in P concentration or in foliar NRE. Increases in MAT increased nutrient return via litterfall for N, K, Mg, and Zn and foliar NUE for Mn and Cu, while decreasing nutrient return for Cu and foliar NUE for K. The N:P of litterfall and live foliage increased with MAT, while there was no detectable effect of MAT on C:P. The ratio of live foliar N or P to base cations and micronutrients was variable across elements and species. Increased MAT resulted in declining N:K and P:K for one species, while only P:K declined for the other. N:Ca and N:Mn increased with MAT for both species, while N:Mg increased for one and P:Mn increased for the other species. Overall, results from this study suggest that rising MAT in tropical montane wet forest: (i) increases plant productivity and the cycling and availability of N, K, Mg, and Zn; (ii) decreases the cycling and availability of Mn and Cu; (iii) has little direct effect on P, Ca or Fe; and (iv) affects ecological stoichiometry in ways that may exacerbate P–as well as other base cation and micronutrient – limitations to tropical montane forest productivity.

## Introduction

Rising global temperatures are predicted to increase biogeochemical process rates including the cycling of carbon (C) (Boisvenue and Running, 2006; Heimann and Reichstein, 2008; Litton and Giardina, 2008; Lin et al., 2010) and nitrogen (N) (Hart and Perry, 1999; Rustad et al., 2001; Bai et al., 2013). Cross-site syntheses (Raich et al., 2006; Litton and Giardina, 2008), eddy flux networks spanning global temperature gradients (Baldocchi et al., 2001; Luyssaert et al., 2007), and elevation gradients (Moser et al., 2011; Giardina et al., 2014; Malhi et al., 2017), generally show that in the absence of water limitations, rising mean annual temperature (MAT) increases forest C cycling. *In situ* soil warming, greenhouse and open-top chamber studies (Rustad et al., 2001; Bai et al., 2013) and soil core replacement studies (Hart and Perry, 1999; Hart, 2006) also collectively show that rising temperatures increase N cycling and availability. Despite this growing body of work, insights into how long-term and whole forest warming affect the suite of essential and potentially limiting nutrients are lacking, and tropical forests are especially understudied despite accounting for a significant proportion of global terrestrial C storage and productivity (Saugier et al., 2001; Bonan, 2008).

Methodological constraints associated with warming an entire forest are the major driver of this knowledge gap. For example, soil warming manipulation experiments (e.g., Melillo et al., 2017) warm the belowground environment, but tree stems and the forest canopy are not subjected to warming, thereby decoupling above- and belowground ecophysiological processes that regulate the supply and acquisition of C, N, other essential nutrients and water (Chapin III et al., 2011). Other studies have addressed this methodological concern by warming single small trees or even very small statured forests (Chung et al., 2013), but these studies have been limited to temperate regions (Cavaleri et al., 2015) and unavoidably represent short-term (<10 year) and small-scale (<0.1 ha) insights into whole forest response to rising temperatures. Moreover, nutrient responses to warming, which are critical to understanding feedbacks to forest C budgets and terrestrial C balance, remain understudied in temperate and especially tropical regions (Melillo et al., 2011).

As a result, confidence in model-based forecasts of the role of forests in mitigating rising atmospheric CO_2_ levels is constrained by the lack of whole ecosystem warming studies. Classic expectations point to increased productivity in a warmer world, but warming can create or exacerbate other limitations to productivity. For example, Free Air CO_2_ Enrichment (FACE) experiments have shown that macro- and micronutrients can respond to CO_2_ enrichment in complex ways (Liu et al., 2007), with initial CO_2_-driven increases in productivity sometimes disappearing as N becomes immobilized and progressively limiting to productivity (Norby et al., 2010; Feng et al., 2015; Terrer et al., 2019). Further, nutrient availability can influence decomposition rates and the release of nutrients from decomposing litter (Liu et al., 2007). Together, these studies highlight that a more complete understanding of the response of forest C cycling to rising temperatures should include assessments of impacts on the cycling and availability of essential macro- and micronutrients.

Evidence from elevation gradient studies indicates that increasing MAT elevates N cycling rates and availability. Marrs et al. (1988) found that field net N mineralization and nitrification rates increased with rising MAT across a 2,600 m elevational gradient in tropical wet forests of Costa Rica. Cardelús and Mack (2010) documented enrichment in canopy tree foliage δ^15^N with increasing MAT along this same gradient, indicating increased N cycling rates (Craine et al., 2009). Across an Ecuadorian gradient, Moser et al. (2011) found that N limitations to productivity increased with elevation, while in an Arizona, United States reciprocal soil core transplant experiment, a 2.5°C increase in MAT resulted in an 80% increase in net N mineralization and nitrification rates (Hart and Perry, 1999; Hart, 2006). Results from gradient studies are supported by many manipulative soil warming studies. The first meta-analysis of 12 studies Rustad et al. (2001) reported that experimental warming increased net N mineralization on average by 46%. In a more recent meta-analysis of 528 observations from 51 studies, Bai et al. (2013) reported that experimental soil warming increased net N mineralization (52%), net nitrification (32%), and pools of soil inorganic N (20%) and leaf N (3%). Neither meta-analysis included tropical sites, but a tropical mesocosm soil warming study documented more than a doubling of extractable soil NO_3_^–^ with soil warming (Liu et al., 2017). The combined results of elevation gradient and manipulative studies demonstrate that in the absence of moisture limitations, warming causes a near universal increase in N cycling and availability. Liu et al. (2017) also reported that soil warming increased soil P availability by ∼20%, but the hypothesis that warming accelerates biogeochemical process rates for other elements has yet to be tested in a whole forest warming study, and the response of essential nutrients other than N and P to whole forest warming is largely unknown (Vitousek et al., 1992; Tanner et al., 1998).

Numerous methods are used to assess nutrient limitation in forests including manipulative (e.g., fertilization experiments), indicator-based (e.g., foliar stoichiometry, nutrient resorption efficiencies), laboratory assay, and nutrient depletion-based methods (Sullivan et al., 2014). None are problem free or equally suited for all ecosystem types, but all have shown utility for assessing nutrient limitation, particularly when multiple techniques are applied in a single study (Sullivan et al., 2014). Aboveground litterfall and nutrient return via litterfall are robust indicators of ecosystem metabolism (Vitousek, 1984; Litton et al., 2007) because aboveground litterfall is a major avenue for C and nutrient transfer between vegetation and soils (Vitousek, 1984; Uriarte et al., 2015), with litterfall varying strongly by species and forest type (Binkley and Giardina, 1998; Tang et al., 2010), soil type (Vitousek, 2004), over time (Chave et al., 2010), and in response to changes in atmospheric CO_2_ concentrations (Norby et al., 2005) and climate (Sayer and Tanner, 2010). Litterfall is also strongly linked to total above- and belowground C flux in forests, accounting for approximately 10% of gross primary production and showing little variation across a global range in forest types (Raich and Nadelhoffer, 1989; Litton et al., 2007).

Elevation gradients in MAT offer an observational approach to monitoring the effects of rising temperatures on whole ecosystems. While avoiding the issues discussed above, gradient-based approaches are often poorly constrained with respect to covariation of other variables that drive ecosystem processes (e.g., moisture availability, soils, vegetation, land-use and disturbance history). We established a highly constrained MAT gradient spanning 13.0 to 18.2°C in mature tropical montane wet forest on Hawai’i Island in which substrate age and soil type, vegetation composition, disturbance history, soil water availability, and solar radiation are all relatively constant (Litton et al., 2007; Giardina et al., 2014; Selmants et al., 2014). We view this MAT gradient as a model study system (distinct from a mesocosm) for testing hypotheses about the effects of rising MAT on ecosystem processes because these fully functioning ecosystems are not constrained by unnecessary variation (Vitousek, 2004). Further, this model study system is: (i) tractable because the entire gradient occurs over a spatial scale of <15 kilometers, greatly facilitating the logistics of conducting regular intensive measurements; (ii) simple because tree diversity in all plots is low and homogenous for tropical forests (Ostertag et al., 2014; Lutz et al., 2018), with the same two species accounting for 84–97% of stand tree basal area in all plots (Litton et al., 2011); and (iii) representative because these forests are structurally and functionally similar to other wet tropical forests despite low tree diversity (Ostertag et al., 2014; LaManna et al., 2017; Johnson et al., 2018; Lutz et al., 2018). For example, Lutz et al. (2018) showed that across the Forest Global Earth Observatory (ForestGEO) network of forest dynamics plots, the tropical montane wet forests from our MAT gradient (Laupāhoehoe) are structurally representative of moist and wet tropical forests with respect to: stand density (Laupāhoehoe = 3925 stems ha^–1^; global mean = 5276; global range = 1692–8956); stand biomass (Laupāhoehoe = 241 Mg ha^–1^; global mean = 305; global range = 111–495); large diameter threshold above which half of total biomass in a forest is contained (Laupāhoehoe = 63 cm; global mean = 47; global range = 29–72); and percentage of total biomass contained in the largest 1% of trees (Laupāhoehoe = 58%; global mean = 57; global range = 17–83). With respect to function, total belowground C flux (TBCF) for our plots (1200 to 1800 g C m^–2^ yr^–1^; Giardina et al., 2014), a robust indicator of belowground function (Litton et al., 2007), is comparable to the range of TBCF reported for tropical forests globally (600 to 1600 g C m^–2^ yr^–1^; Litton and Giardina, 2008).

We used this model study system to examine how aboveground litterfall, live foliar nutrient concentration, foliar nutrient resorption efficiency (NRE), nutrient return via litterfall, and nutrient use efficiency (NUE) of a suite of macronutrients (C, N, P, K) and micronutrients (Ca, Mg, Mn, Cu, Zn, Fe) vary with MAT. To understand MAT effects on ecological stoichiometry, we examined C:N, C:P, and N:P, and the amounts and ratios of K, Ca, Mg, Mn, and Fe, in stand-level litterfall and live foliage of the two tree species that dominate all plots across this gradient. From global data analyses (Raich et al., 2006; Luyssaert et al., 2007; Litton and Giardina, 2008; Giardina et al., 2014) and prior work from our MAT gradient, we know that rising MAT increases: (i) the flux of C in litterfall by increasing net primary production; and (ii) soil NO_3_^–^ availability via increased ammonia oxidizer activity (Pierre et al., 2017).

Based on this prior work, we hypothesized that rising MAT would: (***H1***) increase live foliage concentrations of a suite of macro- and micronutrients due to temperature-driven increases in N cycling and availability (Rustad et al., 2001; Bai et al., 2013; Pierre et al., 2017); (***H2***) decrease NRE of foliar N and P, as increased live foliage nutrient concentrations (i.e., ***H1***) drive decreased foliar resorption of nutrients (Kobe et al., 2005; Vergutz et al., 2012), macronutrient NRE appears to be negatively correlated with MAT across broad latitudinal gradients (Vergutz et al., 2012), and forest productivity appears to be co-limited by N and P in this study system (Vitousek and Farrington, 1997); (***H3***) increase nutrient return through litterfall and decrease NUE for the suite of macro- and micronutrients examined based on meta-analyses results demonstrating temperature-driven increases in the cycling and availability of N (Rustad et al., 2001; Bai et al., 2013); (***H4***) given ***H1*** and ***H3***, not alter ecological stoichiometry in litterfall and live foliage C:N:P (McGroddy et al., 2004); and (***H5***) decrease N:element and P:element for K, Ca, Mg, Mn, Cu, Zn, and Fe based on previous findings that N:element and P:element generally decrease with experimental warming and with increasing MAT across continental scale gradients (Tian et al., 2019).

## Materials and Methods

### Site Description

The study was conducted within nine 20 × 20 m permanent plots arrayed across a 5.2°C MAT gradient ranging from 800 (18.2°C) to 1600 (13°C) m above sea level in the Laupāhoehoe Unit of the Hawaii Experimental Tropical Forest (19°56′41.3″ N, 155°15′44.2″ W) and the Hakalau Forest National Wildlife Refuge (19°50′31.3″ N, 155°17′35.2″ W) on the Island of Hawaii. Mean annual precipitation ranges from ∼3 m at the top of the elevation gradient to ∼4.5 m at the bottom (Supplementary Table S1); because potential evapotranspiration also increases with MAT (Supplementary Table S1), we found very little variation (CV = 10.5%) in mean monthly soil water content across plots – a critical index of water availability to plants (Supplementary Table S2). Because all plots are located below the average base height of the trade wind inversion, the above-canopy light environment varies by <5% across this MAT gradient (Supplementary Table S1). All plots are defined as tropical montane wet forests and classified as mature and mildly aggrading *Metrosideros polymorpha* Gaudich.-*Acacia koa* A. Gray forests (Litton et al., 2011), typical of windward forests on Hawaii Island (Asner et al., 2009; Jacobi et al., 2017). The canopy tree species *M. polymorpha* and the mid-canopy tree species *Cheirodendron trigynum* (Gaudich.) A. Heller comprise 84 to 97% of tree basal area (BA) across all plots (Litton et al., 2011; Selmants et al., 2014).

We used high resolution (<2 m horizontal and <0.2 m vertical) light detection and ranging (LiDAR) to select the nine plots with maximum basal area for a given elevation within the same soil type. These plots were selected from 10 to 20 candidate plots per elevation that were identified as being centered within larger stands of similarly sized forest. Consequently, stand level increases in BA and decreases in stand density with MAT represent real effects of MAT (for a detailed description of plot selection methods see Litton et al., 2011; Selmants et al., 2014). The mildly aggrading, mature condition of plots and the larger surrounding forest was determined using repeat high resolution LiDAR imagery to examine biomass change over time (Kellner and Asner, 2009).

Soils across the gradient are classified as tephra-derived, moderate to well-drained hydrous, ferrihydritic/amorphic, isothermic/isomesic Acrudoxic Hydrudands of the closely related Akaka, Honokaa, Maile, and Piihonua soil series (Supplementary Table S2). These soil series are moderately common across Hawaii Island covering ∼40,000 ha^1^, with the larger Andisol soil order comprising ∼50% of all soils on Hawaii Island (Deenik and McClellan, 2007). Based on radiocarbon analyses of soils to 1 m depth, soil age across all plots is ∼20,000 years (Giardina et al., 2014), one of three common soil ages across windward Hawaii Island. Soil pH, cation exchange capacity, base saturation and bulk density are all relatively constant across plots (Supplementary Table S2).

### Aboveground Litterfall

Fine aboveground litterfall (foliage, reproductive tissue, and branches and twigs < 2.54 cm diameter) was collected monthly from eight replicate 0.174 m^2^ litter traps in each of the nine plots for one year from April 2009 to March 2010 (Giardina et al., 2014). Litterfall from traps within a given plot was composited, resulting in a single litterfall sample per MAT plot for each of 12 months. Litterfall was placed in a forced-air drying oven within 12 h of collection, dried at 70°C to a constant mass, and sorted and weighed by species and component: tree leaf litterfall, tree fern litterfall, reproductive tissue (fruits and flowers), woody material, and other material too fine to identify. Component litterfall for each month and plot was ground on a Wiley-mill and then processed on a ball mill until the entire sample could be passed through a #40 mesh.

### Live Foliage Nutrient Concentration and Nutrient Resorption Efficiency

Live foliage in each plot was collected in 2010 from three individuals of each of the two dominant tree species (*M*. *polymorpha* and *C*. *trigynum*), which together comprise ≥84% of stand tree basal area (Litton et al., 2011). All samples were taken from the most recent fully expanded cohort of leaves from the middle (*M. polymorpha*) or upper (*C. trigynum*) third of the canopy that were exposed to full sunlight at least part of the day. Individual leaves (3–5 from each tree) were oven-dried at 70°C in a forced-air oven to a constant mass and ground on a ball mill to pass a #40 mesh. All nutrient analyses (C, N, P, Mg, K, Ca, Mn, Zn, Cu, and Fe) were conducted at the University of Hawaii at Hilo Analytical Lab. Carbon and N concentrations were measured on a Costech 4010 Elemental Combustion system; other nutrients were measured on a Varian Vista MPX ICP-OES after approximately 0.25 g of sample was dry-ashed (5 h at 500°C) and resuspended in 0.5 M HCl.

Nutrient resorption (or retranslocation) efficiency (NRE), defined as the proportional (%) withdrawal of a nutrient during senescence, was calculated for N and P in both *M. polymorpha* and *C. trigynum* on a mass basis following Vergutz et al. (2012) as:

NRE=1-(MassofNutrientsinFoliarLitterfall/MassofNutrientsinLiveFoliage)×100

Live leaf mass was estimated as the average value for each species at each MAT plot from the live foliage samples (see above). To account for leaf mass loss during senescence, a necessary step for estimates of resorption (van Heerwaarden et al., 2003), we used a global mass loss correction factor of 0.78 for evergreen angiosperms from Vergutz et al. (2012). This approach assumes that leaching of nutrients from litterfall traps between monthly litterfall collections is minimal. We limited this analysis to N and P because prior studies have shown that resorption of these two nutrients is orders of magnitude higher than leaching losses (see Vergutz et al., 2012). Nonetheless, reported NRE values for N and P are likely overestimates. This limits our ability to compare our NRE values with those of other studies, but still allows for exploration of relationships with MAT within our study system, with the assumption that tissue mass loss resulting from senescence does not vary along the MAT gradient – a reasonable assumption given the constrained nature of the gradient.

### Nutrient Return via Litterfall

Litterfall nutrient concentrations of C, N, P, K, Mg, Ca, Mn, Zn, Cu, and Fe were analyzed separately for each plot, month and litterfall component as described above. Across plots and months, nutrient concentrations were determined directly for 89% of all litterfall samples (range of 85–92% for individual plots). For the other ∼10% of litterfall (range of 7–14% across plots), there was not enough tissue available to analyze nutrient concentrations directly, so nutrient concentrations were based on the average of all available values for that plot and litterfall component from the remaining time periods. In so doing, we assumed that there was no variability in litterfall nutrient concentration for a given component at a given MAT over the course of a year. The remaining 1% of total litterfall (range of 0.3–2.4% across plots) was not directly analyzed or estimated for nutrient concentration, and was only included in the analysis of litterfall mass.

Ideally, nutrient use efficiency (NUE; production of organic matter per unit of nutrient taken up) is estimated from total production per total amount of a nutrient acquired by a plant over a given amount of time. Alternatively, nutrient return via litterfall can be calculated as the product of the mass and the nutrient concentration of a given litterfall component for a given MAT and month and summed across components and months for a given plot to calculate NUE following Vitousek (1982) as: NUE = Mass_litterfall_ / Nutrient return via litterfall. This approach defines Mass_litterfall_ (g m^–2^ yr^–1^) as the total litterfall mass for a given plot, with nutrient concentration of litterfall (g m^–2^ yr^–1^) calculated as above. This alternative approach may overestimate NUE for reasons relating to nutrient resorption, such that estimations are sensitive to NRE. Because NRE did not vary with MAT for any nutrient examined (see section “Results” below), we viewed this approach as providing a reasonable index of the response of NUE to MAT (Vitousek, 1982).

### Ecological Stoichiometry

We examined MAT effects on mass-based ecological stoichiometry of C:N:P, as well as the ratios of N and P to K, Ca, Mg, Mn, and Fe, for total annual litterfall and live foliage of the canopy and mid-story dominant trees (*M*. *polymorpha* and *C*. *trigynum*, respectively). Results were used to index relative nutrient availability and nutrient limitation (Sullivan et al., 2014).

### Data Analyses

We used simple linear regression analyses (SPSS Statistics Ver. 25.0; IBM Corp.) to examine MAT effects on the various independent variables. Because most variables met assumptions of normality and homogeneity of variance and these tests are difficult to interpret with small sample sizes, all analyses were conducted on untransformed data. Individual plots were the experimental unit for all analyses (*n* = 9); litterfall collections were composited across traps within months and a given plot to give a single representative subsample of each litterfall component for each plot and month for nutrient content analysis. This approach does not allow for examination of within-plot variability, but a prior study along this MAT gradient showed that the within-plot coefficient of variation (CV) for soil-surface CO_2_ efflux, a highly spatially variable C flux, ranged from 17 to 46%, with an average CV of 33% across all plots and increasing within-plot variability with increasing MAT (Litton et al., 2011). As a result of these small sample sizes, α = 0.10 was used to determine significance, with a *P* ≤ 0.10 cutoff used to present regression lines.

## Results

### Aboveground Litterfall

**FIGURE 1:**
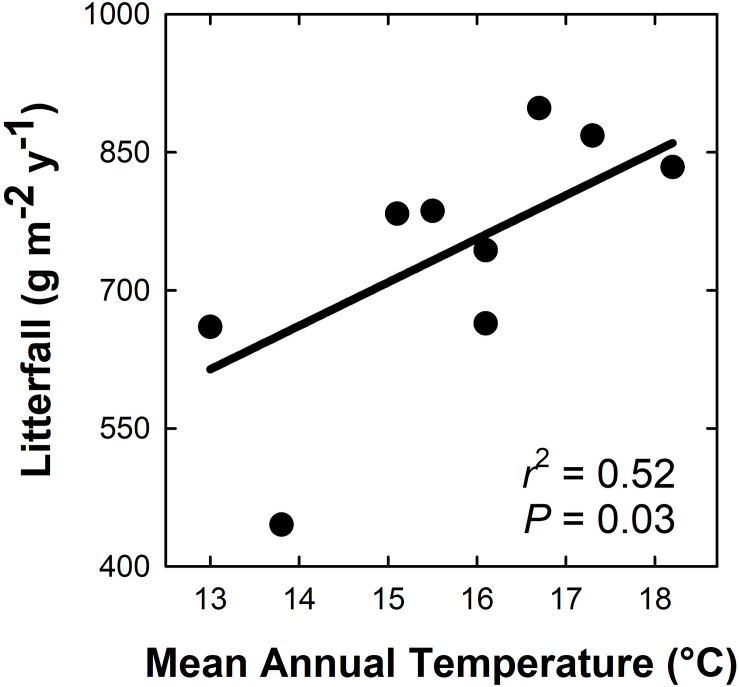
Aboveground litterfall mass (g biomass m^– 2^ yr^–1^) increases with mean annual temperature across a 5.2°C gradient in a Hawaiian tropical montane wet forest.

Aboveground litterfall mass ranged from ∼450 to 900 g m^–2^ yr^–1^ across all plots, and increased linearly and positively with MAT (Figure 1; *r*^2^ = 0.50; *P* = 0.03). For every 1°C increase in MAT, litterfall increased by 47.3 g m^–2^ yr^–1^. *M. polymorpha* foliage was the primary litterfall component across the MAT gradient, accounting for an average of 47% (range of 33–57%) of total annual litterfall across plots. Overall, tree foliage was the primary component of litterfall across plots (47–78% of total litterfall), followed by woody material (10–20%), *Cibotium* spp. fronds (2–19%), unidentified material (3–9%), and reproductive tissues (1–9%).

### Live Foliage Nutrient Concentration and Nutrient Resorption Efficiency

**FIGURE 2:**
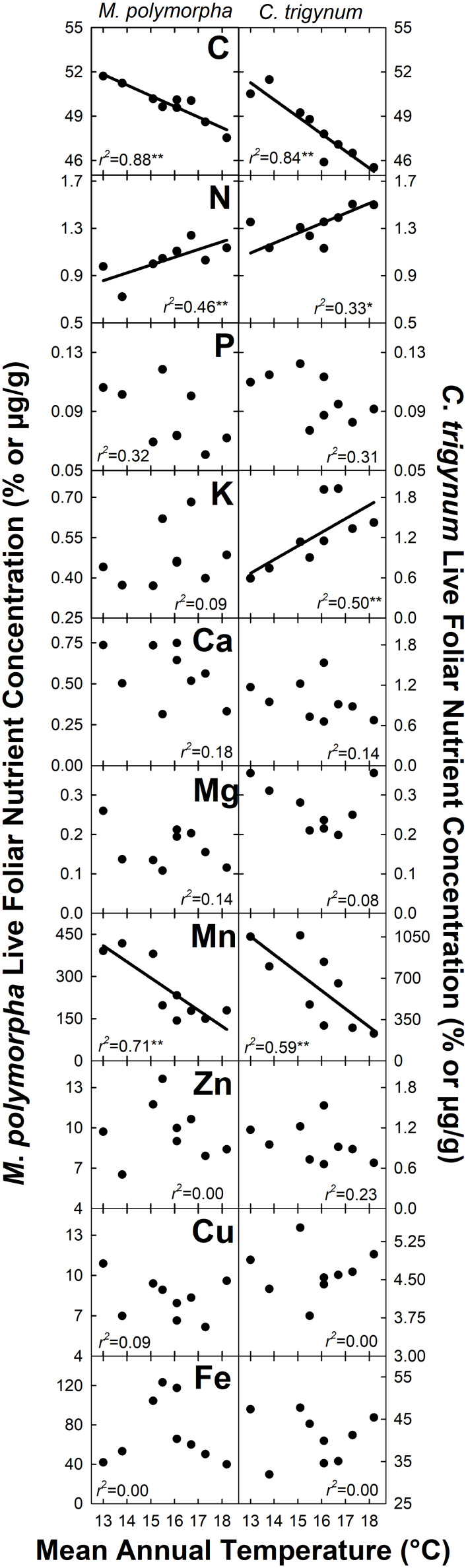
Nutrient concentration of live foliage biomass for the two dominant trees (*Metrosideros polymorpha* and *Cheirodendron trigynum*) in a Hawaiian tropical montane wet forest across a 5.2°C mean annual temperature gradient. Values are displayed as % for macronutrients (C, N, P, K, Ca, and Mg) and ug g^–1^ for micronutrients (Mn, Zn, Cu, and Fe). Significance of regression is indicated as *P < 0.10 and **P < 0.05.

**FIGURE 3:**
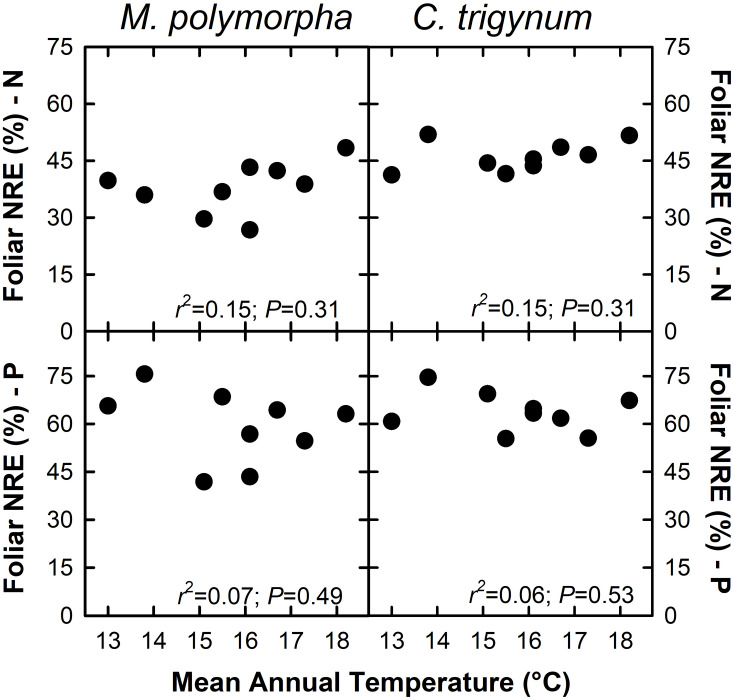
Foliar nutrient resorption efficiency (NRE; %) of nitrogen (N) and phosphorus (P) in the two dominant trees (*Metrosideros polymorpha* and *Cheirodendron trigynum*) does not vary across a 5.2°C mean annual temperature gradient in Hawaiian tropical montane wet forest.

Nitrogen concentration of live foliage increased with MAT for both *M*. *polymorpha* (*r*^2^ = 0.46; *P* = 0.05) and *C*. *trigynum* (*r*^2^ = 0.33; *P* = 0.10) (Figure 2). Live foliar K concentration also increased with MAT for *C*. *trigynum* (*r*^2^ = 0.50; *P* = 0.03), but no detectable effect was observed for *M*. *polymorpha* (*r*^2^ = 0.09; *P* = 0.44). In contrast, live foliar C and Mn concentrations decreased with MAT for both *M*. *polymorpha* (C: *r*^2^ = 0.88 and *P* < 0.01; Mn: *r*^2^ = 0.71 and *P* = 0.01) and *C*. *trigynum* (C: *r*^2^ = 0.84 and *P* < 0.01; Mn: *r*^2^ = 0.59 and *P* = 0.02). There was no detectable effect of MAT on live foliar nutrient concentrations of the other elements for either species (Figure 2), or on foliar nutrient resorption efficiencies for N or P in either *M*. *polymorpha* (*r*^2^ ≤ 0.15) or *C*. *trigynum* (*r*^2^ ≤ 0.15) (Figure 3).

### Nutrient Return via Litterfall

**FIGURE 4:**
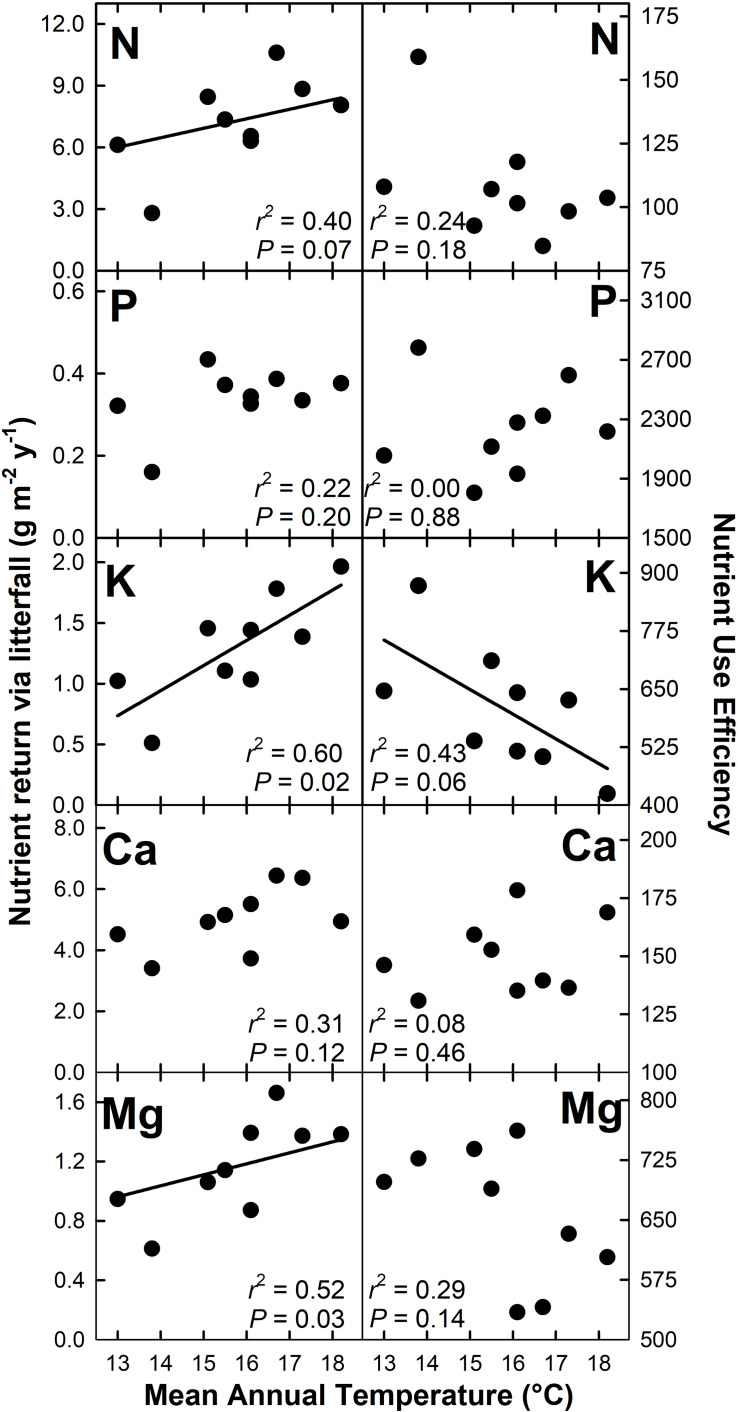
Macronutrient return via total litterfall and macronutrient use efficiency for a Hawaiian tropical montane wet forest across a 5.2°C mean annual temperature gradient.

**FIGURE 5:**
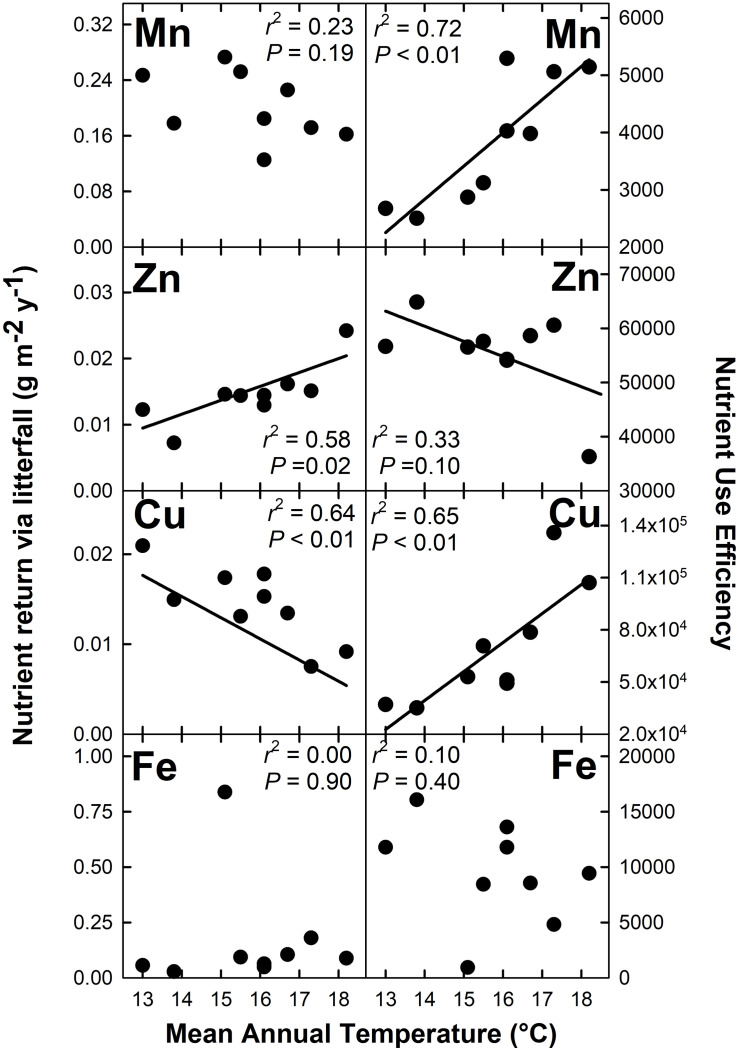
Micronutrient return via total litterfall and micronutrient use efficiency for a Hawaiian tropical montane wet forest across a 5.2°C mean annual temperature gradient.

Nutrient return via litterfall increased with MAT for N (*r*^2^ = 0.40; *P* = 0.07), K (*r*^2^ = 0.60; *P* = 0.02), Mg (*r*^2^ = 0.52; *P* = 0.03), and Zn (*r*^2^ = 0.58; *P* = 0.02) but decreased with MAT for Cu (*r*^2^ = 0.64; *P* < 0.01) (Figures 4, 5). There were no detectable effects of MAT for P, Ca, Mn and Fe (*P* > 0.12). In turn, we found that MAT decreased NUE for K (*r*^2^ = 0.43; *P* = 0.06) and marginally for Zn (*r*^2^ = 0.33; *P* = 0.10), and increased NUE for Mn (*r*^2^ = 0.72; *P* < 0.01) and Cu (*r*^2^ = 0.65; *P* < 0.01). There was no detectable effect of MAT on NUE for the remaining elements examined (*P* > 0.14) (Figures 4, 5).

### Ecological Stoichiometry

**FIGURE 6:**
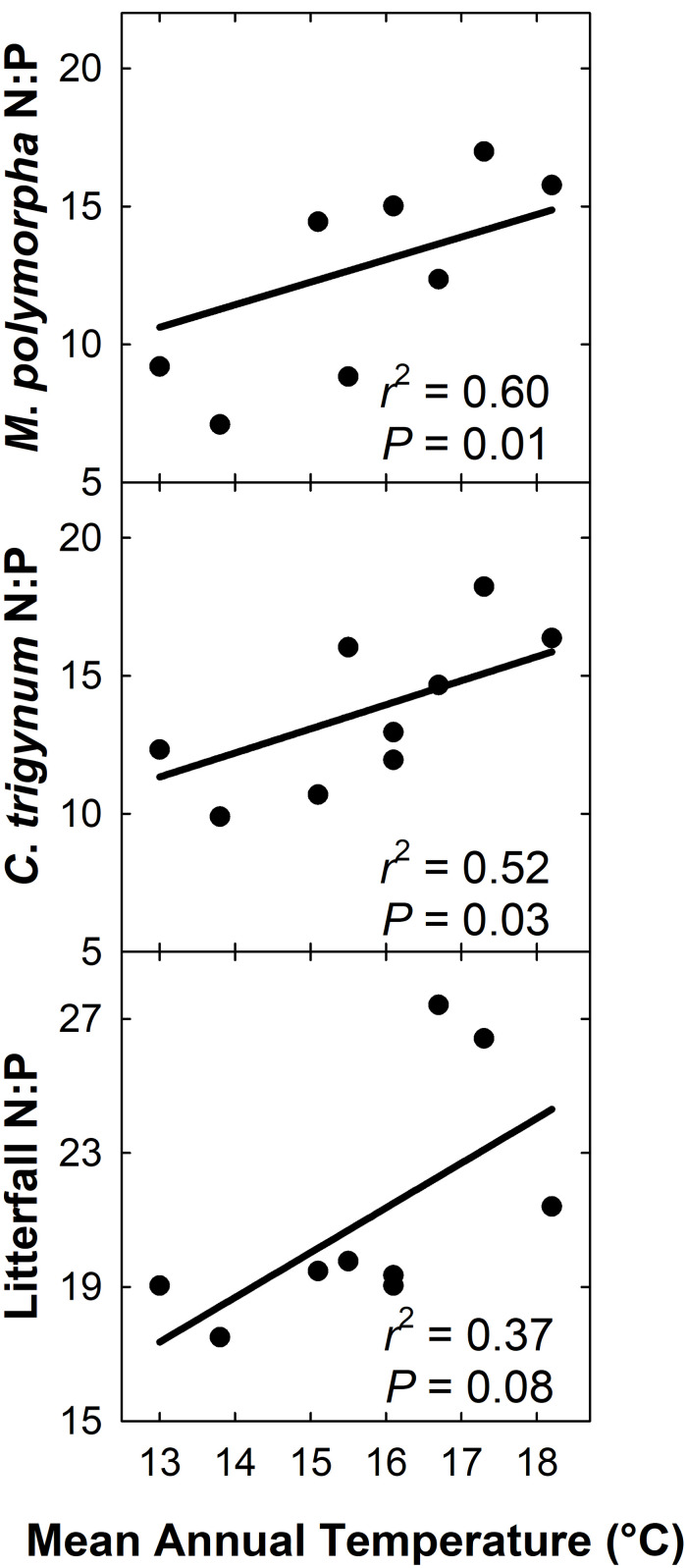
N:P of litterfall and live foliage of both *M*. *polymorpha* and *C*. *trigynum* increased across a 5.2°C mean annual temperature gradient in Hawaiian tropical montane wet forest.

We detected no effect of MAT on litterfall C:N (*r*^2^ = 0.22; *P* = 0.21), but C:N of live foliage decreased with MAT for both *M*. *polymorpha* (*r*^2^ = 0.51; *P* = 0.03) and *C*. *trigynum* (*r*^2^ = 0.54; *P* = 0.02). Litterfall N:P increased with MAT (*r*^2^ = 0.37; *P* = 0.08), as did live foliage N:P for both *M*. *polymorpha* (*r*^2^ = 0.60; *P* = 0.01) and *C*. *trigynum* (*r*^2^ = 0.52; *P* = 0.03) (Figure 6). There was no detectable effect of MAT on either C:P of litterfall (*r*^2^ = 0.01; *P* = 0.81) or of live foliage for either *M*. *polymorpha* (*r*^2^ = 0.27; *P* = 0.16) or *C*. *trigynum* (*r*^2^ = 0.11; *P* = 0.39).

**FIGURE 7:**
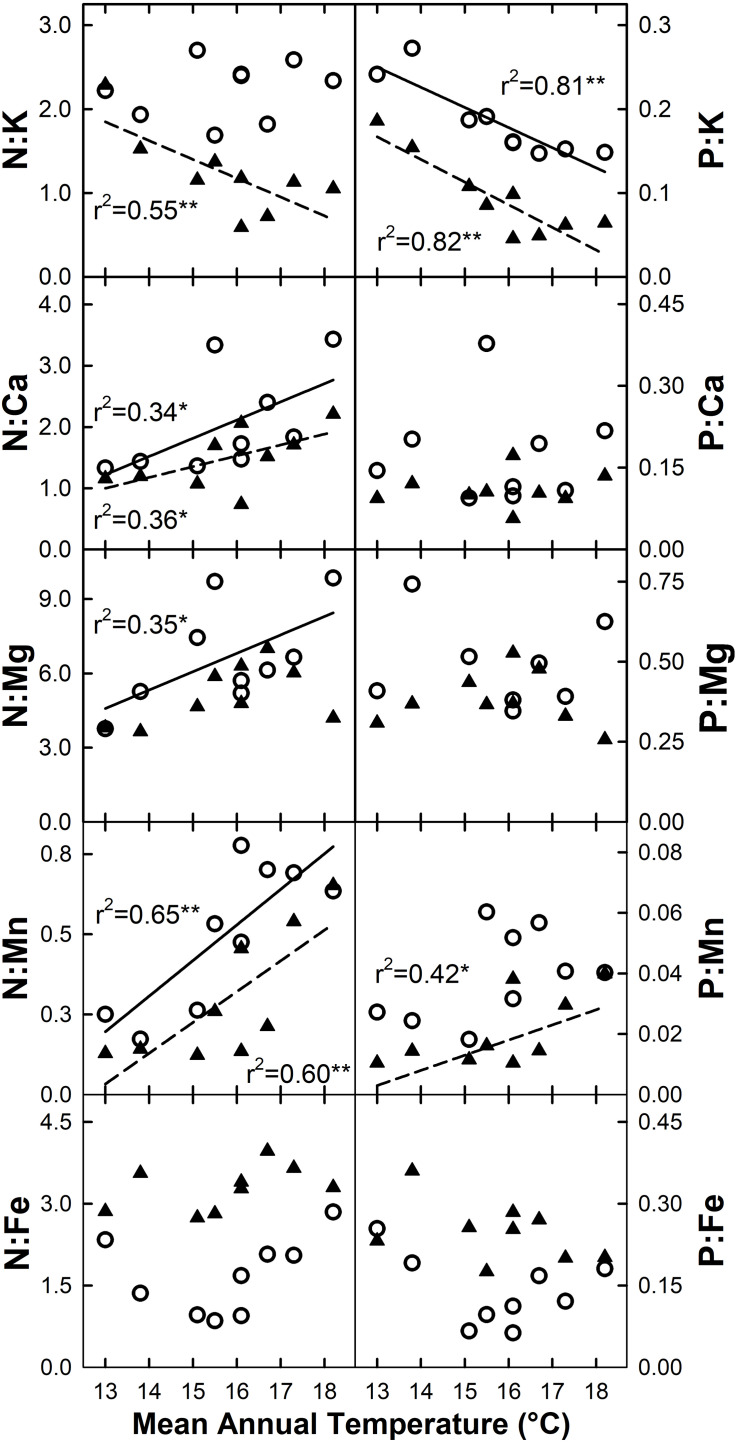
Stoichiometric responses of live foliage nitrogen (N) and phosphorus (P) ratios with micro- and macronutrients for *M*. *polymorpha* (open circles and solid regression lines) and *C*. *trigynum* (closed triangles and dashed regression lines) across a 5.2°C mean annual temperature gradient in Hawaiian tropical montane wet forest. Significance of regression is indicated as *P < 0.10 and **P < 0.05.

The ratio of N or P to base cations and micronutrients for live foliage was variable across elements, and to some extent between species (Figure 7). Increased MAT resulted in declining N:K and P:K for *C*. *trigynum* (N:K *r*^2^ = 0.55, *P* = 0.02; N:P *r*^2^ = 0.82, *P* < 0.01), while P:K declined for *M*. *polymorpha* (*r*^2^ = 0.81; *P* < 0.01). Both N:Ca and N:Mn increased with MAT for *M*. *polymorpha* (N:Ca *r*^2^ = 0.34, *P* = 0.10; N:Mn *r*^2^ = 0.65, *P* = 0.01) and *C*. *trigynum* (N:Ca *r*^2^ = 0.36, *P* = 0.09; N:Mn *r*^2^ = 0.60, *P* = 0.01), while N:Mg increased with MAT for *M*. *polymorpha* (*r*^2^ = 0.35; *P* = 0.09) and P:Mn increased with MAT for *C*. *trigynum* (*r*^2^ = 0.42; *P* = 0.06). The MAT response of these element ratios in litterfall generally aligned with live foliage results, but were more variable and weaker.

## Discussion

Results from our highly constrained MAT gradient demonstrate that warming can: (i) accelerate ecosystem processes; (ii) variably impact the availability of different elements; and (iii) alter ecological stoichiometry. Collectively, these findings provide strong evidence that increased MAT alters the cycling and availability of a broad suite of nutrients in tropical montane forests, with important implications for nutrient limitations to ecosystem processes in a warming world.

### Live Foliage Nutrient Concentration and Nutrient Resorption Efficiency

We found limited support for the hypothesis that increasing MAT would increase live foliage macro- and micronutrient concentrations (***H1***). Only live foliar N concentrations (for both species) and K concentration (for one species) increased with MAT, while C and Mn concentrations showed the opposite pattern, and other nutrients showed no detectable pattern. Across this MAT gradient, increased foliar N concentrations with MAT are likely the result of increased N supply driven by temperature related increases in: (i) litter decomposition rates (Bothwell et al., 2014; Giardina et al., 2014), (ii) NO_3_^–^ cycling and availability via increased ammonia oxidizer activity (Pierre et al., 2017), and (iii) total belowground C investment for nutrient acquisition (Giardina et al., 2014). Accelerated litter decomposition and increased belowground C supply could also explain the higher live foliage K concentration at warmer MATs. While less studied than N or P, fertilization with K has been shown to increase tree growth and plant tissue K concentrations. For example, Tripler et al. (2006) examined a wide diversity of boreal and temperate forests, as well as two tropical mangrove forests, and found that K fertilization increased tree growth in 69% of the forests examined.

Tanner et al. (1998) reported increased live foliar concentrations of N, P, and K with increasing MAT for individual tropical elevation gradients. However, when examined collectively across gradients, foliar N, P and K concentrations were unrelated to MAT. Vitousek (1998) found that for younger soils foliar N was higher at warm compared with cool forests, while Vitousek et al. (1992) found that foliar concentrations of N, P, and K in wet sites generally increased with rising MAT but foliar Ca and Mg showed little variation. Similarly, across a Malaysian elevation gradient, MAT increased leaf litterfall concentrations of N but not those of P, K, Ca, or Mg (Proctor et al., 1989). Bateman et al. (2019) found that for soils in Hawaii with similarly high water balance, total and exchangeable Ca and Mg were low and varied little with MAT. In a global survey, foliar N and P concentrations decreased with increasing MAT (Reich and Oleksyn, 2004). The discrepancy between our results and those of global scale syntheses could be explained by global comparisons capturing much greater variation in environmental drivers of nutrient dynamics other than MAT – for example, soil moisture, substrate and soils, disturbance history, and phylogenetic constraints are difficult to constrain across large scale gradients. Results from global, cross-site syntheses and meta-analyses provide important hypotheses about the impact of rising MAT on biogeochemical processes but may not align with the responses to MAT observed across individual gradients such as ours. As such, our study represents an important test of hypotheses about biogeochemical responses to rising MAT, with results providing much needed local scale data to inform modeling efforts.

We documented a strong, negative, linear response of live foliar Mn concentration to increasing MAT for both species examined (∼14% decrease for every 1°C increase in MAT). Litter decomposition may be strongly controlled by litter Mn content, particularly during late stages of decomposition (Berg et al., 2007, 2010), suggesting that Mn could play a critical role in the cycling and availability of other essential nutrients. However, across our gradient, litter decomposition increased with MAT (Bothwell et al., 2014; Giardina et al., 2014), despite: (i) a steep decline in live foliar Mn concentration; (ii) a trend of decreasing Mn return via litterfall; and (iii) increased nutrient use efficiency for Mn with increasing MAT (see below). These results indicate that Mn availability did not override other controls on litter decomposition rates. Further, while a global analysis indicated that Mn may have a role in controlling plant productivity (Ågren and Weih, 2012), declines in Mn with rising MAT were matched by increased litterfall and TBCF (Litton et al., 2011; Bothwell et al., 2014; Giardina et al., 2014).

We interpret the lack of a detectable response in live foliage nutrient concentration for other elements examined as evidence that either they are not limiting to primary production across our MAT gradient or their availability is not governed by MAT. While productivity appears to be co-limited by N and P in the middle of our MAT gradient (Vitousek and Farrington, 1997), our results support the idea that nutrient limitations are influenced by MAT. In line with these findings, Augusto et al. (2017) concluded that across a global range of sites, N limitations are driven by climate while P limitations are driven by soil parent material–explaining why P availability may be largely unresponsive to climate.

Our NRE estimates for N (38–46%) and P (59–64%) are similar to previously reported values for forests: 47% for N and 54% for P from a global synthesis of woody species (Yuan and Chen, 2009); 56% for N and 58% for P for evergreen woody angiosperms (Vergutz et al., 2012). However, the lack of variation in NRE we observed with MAT contrasts with Vergutz et al. (2012) who reported declining N and P NRE with increasing MAT, and with Yuan and Chen (2009) who reported that N NRE declined while P NRE increased with MAT. While increases in foliar nutrient content appear to drive decreases in foliar resorption (Kobe et al., 2005; Vergutz et al., 2012), Sullivan et al. (2014) found that foliar nutrient resorption was a poor indicator of nutrient limitation along a well-constrained substrate age gradient. Regardless, our results do not support the hypothesis that rising MAT will reduce foliar NRE for N and P (***H2***). Collectively, these findings highlight that because of confounding variation in other drivers of forests processes, global-scale patterns may not predict local responses to rising MAT.

### Nutrient Return via Litterfall

We hypothesized that increased MAT would increase nutrient return through litterfall while reducing nutrient use efficiency (***H3***), which was supported for N, K, Mg, and Zn, but not for Mn and Cu, which showed opposite patterns, or for P, Ca, and Fe, which showed no detectable patterns. This variation across nutrients has important implications for assessing nutrient limitations to productivity and for modeling ecological stoichiometry (see below). Our gradient-based N cycling (Pierre et al., 2017) and litter decomposition (Bothwell et al., 2014) results align with those of short-term warming experiments (Rustad et al., 2001; Bai et al., 2013), and a recent analysis showing a strong effect of MAT on foliar N content across the Hawaii Experimental Tropical Forest where seven of our nine plots are located (Balzotti and Asner, 2018). In contrast, rising MAT had a more variable effect on the biogeochemical cycling of other essential macro- and micronutrients. Tripler et al. (2006) found that among base cations, K cycling was uniquely sensitive to biotic processes. However, we also found that the cycling of Mg and Zn, the later an essential micronutrient involved in a variety of enzymatic reactions (Broadley et al., 2007), both increased with MAT, indicating that elements other than K may be sensitive to warming which is in line with Ågren and Weih (2012) who reported that environmental factors can drive variation in the concentration of foliar macro- and micronutrients.

In our study, Mn and Cu had contrasting responses to MAT compared with N, K, Mg, and Zn, with the availability of each declining with increasing MAT. These declines drove increases in NUE. In contrast, there was no detectable MAT response for P, Ca, and Fe. We speculate that the decreased availability of Mn with rising MAT could indicate that warming accelerates Mn transformation by fungi into insoluble forms (Keiluweit et al., 2015). In contrast we have no interpretation for the decline in Cu availability with rising MAT, nor for the consequences of this response. The lack of detectable response in P availability to MAT can be interpreted as the result of: (i) the stability of P availability with warming, which constrains P uptake as N availability increases with MAT; or (ii) declining availability with rising MAT that is offset by increasing investment by trees belowground (i.e., TBCF) to secure P (Treseder and Vitousek, 2001). The lack of a pattern for Ca and Fe could indicate that declining availability is offset by increasing TBCF, or simply that these elements are not limiting to ecosystem processes.

Nutrient use efficiency (NUE) can serve as a proxy for nutrient availability, and has been shown to be nutrient specific and to vary among plant species (Vitousek, 1982; Tang et al., 2010), and with temperature (Raich et al., 1997; Unger et al., 2010). Our NUE estimates were larger than those found by Tang et al. (2010), but comparable to values reported by Vitousek (1984) and Inagaki et al. (2011). In our study, NUE largely reflected patterns in nutrient return via litterfall, indicating that K, Mg, and Zn availability increase with MAT, whereas Cu and Mn availability decrease with MAT, the later perhaps relating to increased plant demand in response to faster photosynthetic rates (Cu) or decreased solubility with decomposition (Mn).

### Ecological Stoichiometry

Finally, we predicted that increased MAT would not alter ecological stoichiometry of live foliage and litterfall (***H4*** and ***H5***), which was supported only for litterfall and live foliage C:P – likely representing a shift in the potential of N and P to limit productivity. Litter and live foliage C:N and N:P both increased linearly and positively with MAT. These findings point to potential N limitations in the coolest plots, P limitations in the warmest plots and, as previously observed by Vitousek and Farrington (1997), N and P co-limitation in plots in the middle of the MAT gradient. The stronger N:P relationships with MAT for *C*. *trigynum* and *M. polymorpha* live foliage versus senesced litter could relate to: (i) collected litter was composed of diverse tissues (foliage, fruits, small stems) from all species occurring in the plots; (ii) nutrient resorption from live foliage during senescence varies across species and canopy position; (iii) nutrient mobility in response to leaching varies among elements, species and canopy position; and/or (iv) senesced leaves remain attached to stems in the canopy for weeks to months, during which leaching or other forms of decomposition occur.

While interpreting the significance of N:P for nutrient limitations can be complicated (Güsewell, 2004), foliar N:P < 14 generally corresponds with N-limitation, foliar N:P > 16 with P-limitation, and intermediate foliar ratios with N and P co-limitation (Aerts and Chapin, 1999). In our study live foliage N:P for the two study species averaged 9.6 in the two coolest plots, suggesting N limitation to productivity, while N:P for the two warmest plots averaged 16.8, indicating P-limitations. N:P for intermediate plots ranged from 12.4 to 14.0, indicating either weak N-limitation or co-limitation by both N and P. Increased N:P with increasing MAT was also documented in global analyses by Reich and Oleksyn (2004) and Yuan and Chen (2015), the latter of which was interpreted as a decoupling of the N and P biogeochemical cycles under global change. Importantly, ecosystems can adjust to changes in stoichiometry. For example, in Hawaii, Treseder and Vitousek (2001) found that excess N can be used by plants to accelerate phosphatase production and activity under P limiting conditions.

Spatial and temporal variation in macro- and micronutrient availability is an important driver of variation in plant stoichiometry (Ågren and Weih, 2012), with plant stoichiometry arising from physiological constraints that appear to be invariant to factors impacting ecosystem metabolism (McGroddy et al., 2004). While McGroddy et al. (2004) found that forests globally are characterized by relatively well-constrained C:N:P in foliage and litterfall, even small changes in stoichiometry can reflect shifting nutrient limitations. For example, in a global analysis that included data from McGroddy et al. (2004), Zechmeister-Boltenstern et al. (2015) observed that climate exerts a strong influence on the stoichiometry of leaves, roots, and leaf and root litter, with increasing MAT broadly increasing N:P of all four categories. In a more recent synthesis, Augusto et al. (2017) found that climate regulates N limitations, with N availability increasing with warming, but that P limitations are regulated by soil parent material. Working across a highly constrained four million-year geological chronosequence in Hawaii where N and P limitations to productivity vary with soil age, Treseder and Vitousek (2001) found that fertilization altered the mechanisms by which plants acquire soil N and P. For example, N additions stimulated P-tase activity in soil while additions of P suppressed P-tase activity but also reduced mycorrhizal colonization and P uptake capacity. Conversely, Herbert and Fownes (1999) found that variation in soil fertility across the same Hawaii chronosequence had little effect on net primary productivity or allocation of photosynthate to leaves, fine roots and wood.

Interpreting MAT driven changes in the stoichiometry of base cations and micro-nutrients is constrained by the low number of studies that have examined these elements. Tian et al. (2019) identified a negative effect of MAT on foliar N:K, N:Fe and P:Fe across natural climate gradients. Of these, only the decline in N:K aligns with our results, in partial support of ***H5***. Across our plots, foliar N content for *C*. *trigynum* and N return via litterfall both increased with rising MAT, but the increases for foliar and litterfall K were steeper. Because N and K are important macronutrients, their increased availability with warming would indicate that changes to N:K will be less impactful on forest productivity than stoichiometric changes involving P. In contrast to Tian et al. (2019), we also observed increasing K driving declines in foliar P:K for both species examined, also supporting ***H5***. Along with increasing N:P, declining P:K further supports the view that warming may cause or exacerbate P limitations to productivity in these forests.

We showed rising MAT increases N:Ca and N:Mn for both species and N:Mg for *M*. *polymorpha*, which for Ca and Mg were caused by increasing N, and for Mn by increasing N and declining Mn. We also observed increasing P:Mn with rising MAT for *C*. *trigynum*, which was driven solely by a decline in Mn as live foliar P concentrations and P return through litterfall did not vary with MAT. Overall, litter nutrient ratios responded less clearly to increased MAT, perhaps for reasons discussed above. However, litterfall patterns in N:Mn and to a lesser extent P:K and P:Mn were strong, in part because of strong MAT related patterns for N and K (increasing) and Mn (decreasing). Notably, the role of Ca, Mg, and especially Mn in limiting productivity of tropical forests is poorly understood, but across our sites their live foliage concentrations and nutrient return via litterfall declined, remained unchanged, or increased slightly with increased MAT. More information is needed on ecosystem level controls of base cation and micronutrient availability in tropical wet forests.

Taken together, our results indicate that warming in the absence of moisture limitations or altered disturbance regimes has the potential to increase tropical montane wet forest productivity and the cycling and availability of N and K (strong support) and Mg and Zn (moderate support), but will decrease the cycling and availability of Mn and Cu (moderate support). We found little evidence that warming will affect the cycling and availability of P, Ca or Fe, but warming-related increases in the supply of other elements may cause P, or possibly base cation, micronutrient limitations to ecosystem processes. The results presented here provide an enhanced picture of the response of a variety of micro- and macronutrients to future increases in temperature in tropical montane wet forests, which can be used to refine and parameterize ecosystem models needed to forecast ecosystem response to global environmental change.

## Data Availability Statement

The datasets generated for this study are available on request to the corresponding author.

## Author Contributions

CL and CG designed the research, secured the funding, and collected the data. CL, KF, and PS performed the data analyses. CL and CG led interpretation and writing of the manuscript, with assistance from all other authors.

## Conflict of Interest

The authors declare that the research was conducted in the absence of any commercial or financial relationships that could be construed as a potential conflict of interest.

## Footnotes

1 https://soilseries.sc.egov.usda.gov/
