# Supplementary material for: Impact of Mean Annual Temperature on Nutrient Availability in a Tropical Montane Wet Forest
Source: Front Plant Sci. 2020 Jun 12;11:784. doi: 10.3389/fpls.2020.00784 (PMC7304228; doi:10.3389/fpls.2020.00784)
Supplement: Supplementary file 1 [file Table_1.DOCX]

Table S1. Environmental variables for the nine tropical montane wet forest plots along a 5.2°C mean annual temperature gradient on the Island of Hawaii. VWC is volumetric water content and PET is potential evapotranspiration. Data originally published in Selmants *et al.* (2014).

| Mean annual temperature (°C) | Elevation (m) | Mean annual rainfall (mm y^-1^)^a^ | Mean annual PET  (mm y^-1^)^b^ | Mean annual solar radiation  (W m^-2^ y^-1^)^b^ |
| --- | --- | --- | --- | --- |
| 18.2 | 800 | 4570 | 2298 | 201.10 |
| 17.3 | 934 | 4292 | 2232 | 200.86 |
| 16.7 | 1024 | 3975 | 2214 | 202.42 |
| 16.1 | 1116 | 3734 | 2127 | 204.89 |
| 16.1 | 1116 | 3433 | 2137 | 210.06 |
| 15.5 | 1204 | 3181 | 2211 | 214.45 |
| 15.1 | 1274 | 3101 | 2234 | 216.16 |
| 13.8 | 1468 | 4119 | 1888 | 202.63 |
| 13.0 | 1600 | 3282 | 1961 | 213.14 |

^a^Mean annual rainfall estimates from the Rainfall Atlas of Hawaii (Giambelluca *et al*. 2013)

^b^Mean annual potential evapotranspiration and solar radiation estimates from Giambelluca *et al*. (2014)

Table S2. Soil properties for the nine tropical montane wet forest plots along a 5.2°C mean annual temperature gradient on the Island of Hawaii. ECEC is estimated cation exchange capacity and *T*_SOIL_ is soil temperature. Data originally published in Litton *et al.* (2011).

| Mean annual temperature (°C) | Soil Series^a^ | pH^b^ | ECEC^b^  (cmol kg^-1^) | Base Saturation^b^  (%) | Bulk Density^c^  (g cm^-3^) | C Content^c^ (%) | Mean annual  *T*_SOIL_  (°C) | Mean annual Soil VWC  (%) |
| --- | --- | --- | --- | --- | --- | --- | --- | --- |
| 18.2 | Akaka | 4.1 | 9.3 | 57.8 | 0.21 | 9.7 | 18.0 | 55.3 |
| 17.3 | Akaka | 4.2 | 9.6 | 37.8 | 0.19 | 15.7 | 17.3 | 54.8 |
| 16.7 | Akaka | 3.7 | 11.4 | 27.6 | 0.19 | 15.1 | 16.3 | 56.9 |
| 16.1 | Akaka | 3.8 | 16.4 | 27.7 | 0.20 | 15.3 | 15.9 | 48.0 |
| 16.1 | Honokaa | 3.6 | 15. 9 | 35.6 | 0.26 | 13.9 | 15.6 | 50.8 |
| 15.5 | Honokaa | 3.7 | 12.1 | 25.3 | 0.23 | 15.3 | 15.5 | 40.2 |
| 15.1 | Maile | 3.9 | 12.3 | 24.4 | 0.22 | 13.1 | 14.9 | 50.6 |
| 13.8 | Akaka | 4.2 | 8.5 | 27.1 | 0.18 | 15.4 | 13.6 | 55.3 |
| 13.0 | Piihonua | 4.1 | 11.3 | 27.9 | 0.23 | 12.6 | 12.6 | 57.4 |

^a^Soil in all plots classified as hydrous, ferrihydritic/amorphic, isothermic/isomesic Acrudoxic Hydrudands.

^b^pH, ECEC and base saturation quantified on fresh soil samples (10 cores plot^-1^) at field moisture collected to a depth of 10 cm.

^c^Bulk density and carbon content of <2mm mineral soil to a depth of 91.5 cm (3-4 cores plot^-1^).
